# The Diet Quality of Food-Insecure Australian Adults—A Nationally Representative Cross-Sectional Analysis

**DOI:** 10.3390/nu14194133

**Published:** 2022-10-05

**Authors:** Rebecca Lindberg, Sarah A. McNaughton, Gavin Abbott, Christina M. Pollard, Amy L. Yaroch, Katherine M. Livingstone

**Affiliations:** 1 Institute for Physical Activity and Nutrition (IPAN), School of Exercise and Nutrition Sciences, Faculty of Health, Deakin University, Geelong, VIC 3220, Australia; 2 Curtin School of Population Health, Faculty of Health Sciences, Curtin University, Perth, WA 6102, Australia; 3 Enable Institute, Faculty of Health Sciences, Curtin University, Bentley, WA 6102, Australia; 4 Gretchen Swanson Center for Nutrition, Omaha, NE 68154, USA

**Keywords:** food security, diet quality, nutrition inequities, adults, nationally representative survey

## Abstract

Poor diet quality exacerbates risks for acute and chronic conditions. People experiencing food insecurity have an increased likelihood of lower diet quality; however, this has not been investigated in the Australian context. The aim of this cross-sectional study was to examine whether the diet quality of Australian adults differed according to their household food security status. Data were analysed from a nationally representative sample (≥19 years; *n* = 9115) collected as part of the National Nutrition and Physical Activity Survey 2011-12. Household food security status and socio-demographic and health characteristics were assessed using data from an 18-module health interview. A 24 h dietary recall was used to estimate food and nutrient intakes and to calculate the Dietary Guidelines Index (DGI). DGI is a food-based score (0 to 130) that assesses adherence to the 2013 Australian Dietary Guidelines. Survey-weighted linear regression models, adjusted for age and sex, were used to examine diet quality (total DGI and component scores), and total energy and nutrient intake by food security status. Adults from food-insecure households had a mean total DGI score 3.5 points lower (95% CI −5.57, −1.46) than food-secure adults (*p* = 0.001). Adults from households experiencing food insecurity, when compared to those who were food-secure, had several lower DGI component scores including for dietary variety (1.6 vs. 2.3, *p* = 0.009), fruit (3.8 vs. 5.0, *p* = 0.001) and vegetables (3.7 vs. 4.4, *p* = 0.010). Adults from food-insecure households consumed on average more carbohydrates (45.6 vs. 43.3, *p* = 0.004) and total sugar (21.8 vs. 19.0, *p* = 0.003) as a percentage of daily energy and less protein (18.5 vs. 17.2, *p* = 0.004), mono-unsaturated fats (11.2 vs. 11.8, *p* = 0.026) as a percentage of daily energy, and fibre (20.1 vs. 23.0, *p* = 0.003), than food-secure adults. Sub-optimal diet may be one of the contributing factors to, or outcomes of, poorer health in food-insecure populations. Food security interventions are required to better address nutrition in food-insecure households and should be tailored to the health and socio-demographic characteristics of this population.

## 1. Introduction

Due to the impact of the COVID-19 pandemic, the prevalence of household food insecurity is globally on the rise, including in developed economies [1]. This health and social problem that affects households can be both a precursor to, and a consequence of, economic marginalisation and poor health. Food insecurity occurs when there is inadequate access to safe, culturally appropriate, and nutritious food [2]. According to the 2017–2019 estimates by the Food and Agriculture Organization (FAO), the average prevalence of food insecurity across high-income countries was 7.5% and 13.5% in Australia [3], with these rates likely to have risen since then.

Evidence suggests that the diet quality of people experiencing food insecurity can be sub-optimal. A systematic review of 26 diet quality studies from the United States (US) (published 1997–2012) [4] found that adults experiencing food insecurity consumed fewer vegetables, fruit, and dairy products and had lower intake of vitamins A and B-6, zinc, calcium and magnesium when compared to food-secure adults. However, associations between food security status and other aspects of dietary intake (e.g., macronutrients, grains, iron) were not substantiated. In 2014, a nationally representative cross-sectional analysis from the U.S. found that, compared with food-secure adults, food-insecure adults reported a 2-unit lower Healthy Eating Index score [5] and low-income food-insecure adults had a higher consumption of high-fat dairy products, salty snacks, sugar-sweetened beverages, processed meat and consumed fewer vegetables than low-income food-secure adults [6]. Similarly older Americans (aged > 60 years) experiencing food insecurity, when compared to their food-secure peers, have a lower mean total Healthy Eating Index score [7]. A nationally representative study from France [8] found that people experiencing food insecurity consumed less fruit, vegetables and fish than those who were food-secure. In Australia, a study using the National Nutrition and Physical Activity Survey (NNPAS) 2011-12 found that, when compared with the least socio-economically disadvantaged group, the most disadvantaged group had a 2.5–4.5 units lower Dietary Guidelines Index score. Considering that socio-economically disadvantaged populations are more likely to experience food insecurity, an examination of food security status and diet quality in an Australian setting may further highlight priority populations for public health intervention. 

The aim of this cross-sectional study was to examine the diet quality of Australian adults according to household food security status using the nationally representative NNPAS 2011-12. A secondary aim was to investigate the health and socio-demographic characteristics of Australian adults according to food security status.

## 2. Materials and Methods

### 2.1. Study Design and Participants

This manuscript describes a cross-sectional observational study using data from the most recent nationally representative Australian nutrition survey, the NNPAS 2011-12 component of the Australian Health Survey [9]. This survey [10,11] was administered by the Australian Bureau of Statistics (ABS) and sampled households across all states and territories. The NNPAS did not include participants who are Indigenous, as these respondents participated in the National Aboriginal and Torres Strait Islander Nutrition and Physical Activity Survey 2012-13 [12]. Full details of the Australian Health Survey study design are published elsewhere, including in a number of previously published nutrition studies [10,11].

**Figure 1:**
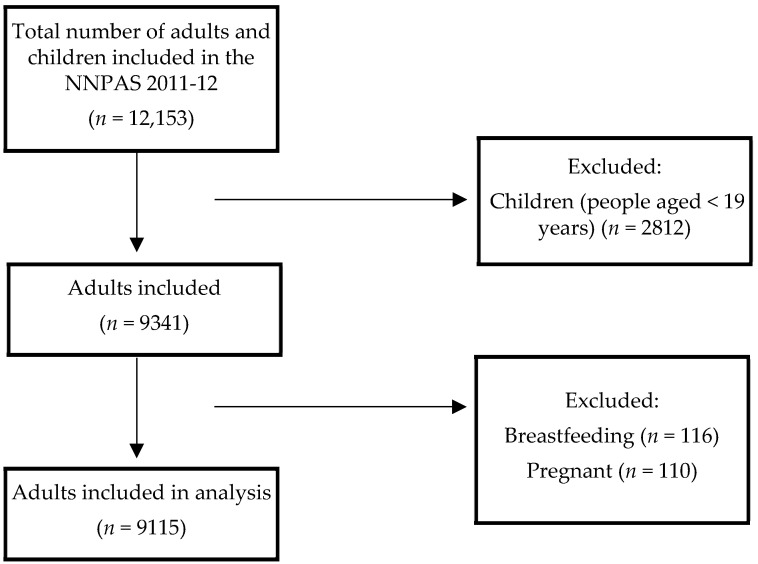
Flow diagram of participants included in the analysis of the National Nutrition and Physical Activity Survey 2011-12.

A total of 14,363 private dwellings were initially selected to participate in the survey, of which 9519 households responded to the NNPAS interview component (77% response rate; *n* = 12,153 adults and children). The interview included 18 modules, on a range of demographic, nutrition, physical activity and health questions. A total of 9341 adults (≥19 years) completed the food security questions in the interview and one 24 h dietary recall (see Figure 1). After excluding pregnant and breastfeeding adults, 9115 adults were ultimately included in the analysis.

### 2.2. Procedures

#### 2.2.1. Food Security

Household food security status was established using a standard single-item measure common in national ABS surveys [9]: asking respondents if they (or any member of the household) had run out of food in the last 12 months and could not afford to buy more (binary variable; yes, no). Affirmative responses were considered ‘food insecure’.

#### 2.2.2. Dietary Intake

Information on consumed food and beverages was collected using a 24 h dietary recall based on the United States Department of Agriculture’s Automated Multiple-Pass Method (adapted to the Australian context) and facilitated by face-to-face trained interviewers. Energy and nutrient intakes (total energy—KJ), percentage energy from total fat, saturated fat, trans fat, mono-unsaturated fat, poly-unsaturated fat, carbohydrates, total sugars and protein, and fibre density and sodium density(g/MJ) were estimated from the recall using the Australian Food and Nutrient Database 2011–2013 [13]. Brief questionnaires were used to report on the usual daily use of salt (both at the table and during cooking, with response options: “not used”, “rarely”, “occasionally” or “very often”) [9].

#### 2.2.3. Diet Quality

Diet quality was assessed by the Dietary Guideline Index (DGI) (see Supplemental Table S1). The DGI is a food-based score that assesses compliance with age- and sex-specific recommendations outlined in the current (2013) Australian Dietary Guidelines [14]. Dietary intakes were scored according to recommended dietary components (food variety, fruit, vegetables, cereals, meat and alternatives, dairy and alternatives, and fluid intake in terms of total consumed beverages and proportion of these that are water) and adverse dietary components (discretionary foods, saturated fat, unsaturated fat, added salt, added sugars, and alcohol). Each component was scored out of 10, with proportionate scoring used to score intakes that fell between the maximum and minimum scoring criteria. For example, if a participant consumed 1 serving of fruit per day, they received a score of 5 out of 10. The total DGI scores range between 0 and 130. A higher score indicates a better diet quality.

#### 2.2.4. Sociodemographic and Health Characteristics

Collected information on sociodemographic and health characteristics included age (discrete; years), sex (binary; male, female), household income based on the gross weekly combined equivalised income of all household members aged ≥15 years (categorised into deciles), educational obtainment assessed on highest level completed (categorised into low—incomplete high school or less; medium—completed high school or incomplete high school and/or certificate/diploma; or high—tertiary qualification), location (determined by using the Australian Statistical Geography Standard Remoteness areas [9] and categorised into major cities of Australia, inner regional Australia and a combined outer regional Australia, remote Australia and very remote Australia), country of birth (categorised into Australia; English-speaking country outside of Australia; non-English speaking countries outside of Australia), marital status (binary; married, not married) and smoking status (categorised into current; ex-smoker; never smoked). Weight (kg) and height (m) were measured by trained interviewers using digital scales to measure weight and a stadiometer to measure height. BMI was calculated from weight and height and categorized into underweight (BMI ≤ 20 kg/m^2^), normal weight (BMI ≤ 24.9 kg/m^2^), overweight (BMI ≥ 25 kg/m^2^ and < 30 kg/m^2^) and obese (BMI ≥ 30 kg/m^2^) [9].

### 2.3. Analysis

Statistical analysis was performed using Stata [15] applying survey weightings to account for the survey design and for the probability of selection. *p*-values < 0.05 were considered statistically significant. As there were no participants with substantive missing data for outcomes or covariates (i.e., no participant had more than 2 variables missing), no exclusions were made on this basis. Multiple imputation by chained equations (50 imputations), with all study variables used in the imputation models, was used for handling missing data, as 7176 adults had complete data for study variables, 1033 were missing BMI, 602 were missing income, and 304 were missing both BMI and income.

Descriptive statistics (means and proportions with 95% CIs) were used to report the distribution of socio-demographic and health characteristics and diet quality according to food security status. Survey-weighted, multiple imputation linear regression models were fitted to test differences in diet quality (DGI total score and component scores; continuous dependent variables), total energy and nutrient intakes (continuous dependent variables) according to food security status (binary independent variable). Confounders were established a priori based on the previous literature [6,16,17]. Data were analysed using the following two covariate models: model 1 (minimally adjusted), included age (continuous) and sex (binary) as covariates; model 2 provided a sensitivity analysis (see supplement 2) and was adjusted for model 1 covariates (age, sex), as well as equivalised household income (categorical), educational obtainment (categorical), country of birth (categorical), marital status (categorical) and smoking status (categorical).

### 2.4. General Procedure

This project (number 2021-357) was declared exempt from ethical review by the Deakin University Human Research Ethics Committee. The STROBE-nut checklist [18] was used to design and report the findings.

## 3. Results

### 3.1. Socio-Demographic and Health Characteristics of Australian Adults by Food Security Status

The survey-weighted estimates indicate most adult participants (95.6%) were assessed as living in households that were food-secure, and 4.4% lived in households experiencing food insecurity. Survey-weighted participant demographic and health characteristics according to food security status are described in Table 1. Adults in food-insecure households were younger (mean age 39.9 vs. 46.9 years) with a higher proportion of being female (61.6% vs. 48.9%) and living below the poverty line (44.7% vs. 18.1%), compared to adults not experiencing food insecurity. Half of those who were food-insecure were employed; 9.4% were unemployed; and 40.9% were not in the labour force, compared with 67.7%, 2.3% and 30.0%, respectively, for food-secure adults. Higher proportions of food-secure adults were married (60.1% vs. 33.8%), and adults experiencing food insecurity reported higher rates of current smoking (50.3% vs. 16.8%). More than one-third (37.9%) of those experiencing food insecurity reported low educational attainment (25.4% of food-secure), and 14.2% had obtained a tertiary qualification (25.5% of food-secure). There were no significant differences according to food security status in terms of country of birth, location or BMI category.

### 3.2. Diet Quality Assessment Using the Dietary Guidelines Index (DGI)

The total DGI mean score for adults in food-secure households was 76.8 (95% CI 76.3, 77.3), and it was 72.8 (95% CI 70.5, 75.2) for those experiencing food insecurity. After adjustment for age and sex (model 1), adults in food-insecure households had a 3.52 (−5.57, −1.46) lower mean DGI score than food-secure adults (*p* = 0.001) (Table 2). In the fully adjusted model, (model 2) the difference between the groups was no longer statistically significant and was closer to half a point (B = - 0.45; 95% CI −2.48, 1.58, see Supplement Table S2). Food variety scores were significantly different (in both models 1 and 2) between the groups, with adults in food-secure households achieving a mean score of 2.3 and food-insecure achieving 1.6. In terms of fruit, vegetables, cereals (including wholegrains), and meat and alternatives, adults in food-insecure households had lower mean scores compared to food-secure adults, with significant differences for model 1 (but not model 2). The mean scores were comparable for dairy and fluid intake. In terms of mean scores for adverse dietary components (discretionary foods, saturated fat, unsaturated fat, added salt, added sugar, and alcohol), the groups were comparable on most scores except moderating unsaturated fat intake, where food-insecure adults obtained higher scores (8.7 vs. 8.2) and, limiting added salt at the table, where food-insecure adults obtained lower scores (2.8 vs. 3.3).

### 3.3. Energy and Nutrient Intakes by Food Security Status

As shown in Table 3, adults in food-insecure households had lower overall energy intake (8147 kJ/day; food-secure 8694 kJ/day, *p* = 0.048), protein intake (17.2% E/day; food-secure 18.5% E/day, *p* = 0.004), mono-unsaturated fat intake (11.2%E/day; food-secure 11.8%E/day, *p* = 0.026), and fibre intake (20.1 g/MJ; food-secure 23.0 g/MJ, *p* = 0.003). Conversely, they had a higher carbohydrate intake (45.6% E/day; food-secure 43.3% E/day, *p* = 0.004) and total sugar intake (21.8% E/day; food-secure 19.0% E/day, *p* = 0.003) than those in food-secure households. After adjusting for age, sex, and additional co-variates equivalised household income, education, country of birth, marital status and smoking status (model 2), significant differences remained between adults in food-secure and -insecure households in terms of protein, mono-unsaturated fat, carbohydrates and total sugar intake (Supplement Table S3).

## 4. Discussion

This is the first study to examine diet quality according to household food security status in Australia. The results suggest differences in the quality of diets and the demographic and health characteristics between Australian adults living in households experiencing food insecurity and those from food-secure households. Adjusted for age and sex, food-insecure adults had more than a three-point lower average total DGI score than their food-secure peers; lower fruit, vegetable, and cereal intake; and lower total energy, protein, fibre, and mono-unsaturated fat intake. Half of the adults in food-insecure households were employed, and one-third had not completed high school (categorised as low educational attainment). Adults in food-insecure households were younger, with a higher proportion of those who were female, unmarried, and living below the poverty line, compared to food-secure adults. In terms of health status, BMI was similar between the two groups, and a larger proportion of adults living in food-insecure households reported being current smokers.

This investigation reports similar findings to national and international research regarding the diet quality of adults residing in food-insecure households. Whilst the diet quality measures have similar components but different scales (in the US, scores range from 0 to 100; in Australia, they range from 0 to 130), a 2014 US investigation [5] found an average of 2 units lower for adults in food-insecure households versus food-secure households, similar to our finding of a 3.5 units difference in the total DGI score. The difference in the Australian scores is equivalent to daily food intakes of one less serving of vegetables or more frequent salt use. These differences, if sustained, could adversely impact diet-related chronic disease risk, such as cardiovascular disease, for food-insecure populations [19].

Higher carbohydrate, lower dietary diversity score, and lower meat (or alternative) intakes are consistent with the literature describing the coping strategies of people who experience food insecurity. Australian qualitative research examining the experience of people on low incomes or in poverty identifies coping strategies aiming to bulk out diets by adding cheaper (often less nutritious) and filling foods, such as low-cost cereals in the daily-food practices of adults reliant on charitable food [20,21]. Both meat and dairy have also been identified in research as more expensive components of a diet that are sometimes forgone by single mothers [22], urban households [23], and households impacted by the COVID-19 pandemic [24]. Consistent with our findings, adults living in food-insecure households in Australia and other high-income countries eat less than their recommended amount of fruit and vegetables and less than their food-secure peers [4,8]. Furthermore, a lower food variety score and higher carbohydrate intake in food-insecure households remained following the adjustment for model 2, suggesting these dietary practices persist independent of strong and inter-connected predictors of food security.

Non-dietary risk factors for chronic disease, such as smoking and high-BMI may be compounded by food insecurity in households. In this study, differences in BMI were not significant overall, although greater proportions of adults who were food-insecure were underweight or obese. There is growing global evidence to suggest there is a paradoxical association between food insecurity and obesity [25]. In our study, the increased likelihood of food-insecure adults currently smoking is also reported in US studies [26]. This further underscores the complex potential syndemic of health, social, and economic factors faced by adults residing in food-insecure households. Reducing household food insecurity could be an effective way to prevent chronic disease, and clinicians are increasingly urged to consider and screen adults [25] and children [27] for food insecurity.

There are several potential research, policy, and practice implications of this study. To our knowledge, this research is a first in Australia and the only study to include detailed dietary data from non-Indigenous adults living in food-insecure households. It provides a methodology that can be repeated and improved upon in the forthcoming ABS nutrition and physical activity survey (planned for 2023) and for ongoing monitoring.

In terms of policy, Australians experiencing food insecurity are more likely to be in receipt of social assistance payments than the general population [28]. Increasing social protection incomes to a level that at least meets the minimum costs of living [28] and ensures the affordability of a nutritious diet is included in those calculations, would help to address some of the concerning dietary intake identified in this study. For Australians experiencing food insecurity whilst in paid employment (49.7% of our sample), the adequacy of wages is important. This study found that the dietary intake of several recommended food groups was sub-optimal in food-insecure adults and that particular sub-populations might be at an increased risk, for example women, people out of the workforce, people who are single, and/or people living below the poverty line. The Government should consider the potential impact of fiscal policies, such as the good and services tax (GST), on these groups, and single-parent households (for example) are more likely to experience food stress with proposed changes to the GST [29,30]. This research supports the calls for a national integrated food and nutrition policy and monitoring and surveillance system that includes a focus on equity, household food security, and priority populations at increased risk of diet-related diseases [31,32].

Practitioners and programmes supporting food-insecure households could sensibly prioritize fruit, vegetables, wholegrains, increased variety, and foods high in mono-unsaturated fats and fibre to improve diet quality. Interventions should seek to target populations identified as at higher risk (e.g., women, single people, and people living below the poverty line). The current findings also emphasize the importance of nutrition and food security interventions for people who smoke.

There are several design strengths and limitations of this study. The ABS 2011-12 NNPAS is a nationally representative study, which is a strength. However, this is the latest national dietary survey that is a decade old, and dietary practices are likely to have changed over that time. The single-item food insecurity measure used is known to under-report [33] and the prevalence among the Australian population may be closer to 10–13% [3], and higher still in Aboriginal and Torres Strait Islander populations [12], and especially since the COVID-19 pandemic [1]. This would suggest that the dietary implications demonstrated by this current study are likely to be reaching further into society. The sample for this analysis was limited to 399 non-Indigenous adults, and despite the sampling strategy to recruit a nationally representative participant group and the use of survey-weighting in the statistical analysis, it is unlikely to represent the diets, health, and demographics of all food-insecure Australian adults and it excludes first-nation people who are disproportionally affected by food insecurity. Unlike the measures used elsewhere [34], the Australian food and nutrition monitoring system does not allow for the assessment of the severity of food insecurity or impact on households with children [35]. Furthermore, in order to maximise the sample size, only one day of dietary recall was used, rather than two days. While this has limitations in terms of assessing the usual intake of individuals, it is appropriate for population monitoring and surveillance [36]. Self-reported dietary assessment is known to be subject to under-reporting; however, 24 h recalls are shown to be the least impacted by measurement error compared to other approaches. The food frequency questionnaire and the tool used in Australia was based on the standardised Automated Multiple Pass method [36,37]. Due to the cross-sectional design, we were unable to infer any temporal relationships between diet and food security status.

## 5. Conclusions

This cross-sectional study examined whether the diet quality of Australian adults differed according to their household food security status. Adults residing in households experiencing food insecurity were found to have a mean total DGI score 3.5 points lower than those in food-secure households. In addition, adults experiencing food insecurity, when compared to those who were food-secure, had lower scores for dietary variety, fruit, and vegetables and consumed more carbohydrates and total sugars and less protein, mono-unsaturated fat, and fibre. If these dietary patterns persist, Australians from food-insecure households are at increased risk of diet-related chronic disease, and thus should be a priority population for public health interventions.

## Figures and Tables

**Table 1:** Survey-weighted socio-demographic and health characteristics of Australian adults by food security status using the NNPAS 2011-12.

Characteristic		Food-Secure (95.6%)	Food-Insecure (4.4%)	*p* Value ^
Age, mean	Years	46.9 (46.8, 47.1)	39.9 (38.8, 41.6)	<0.001
Sex, %	Male Female	51.1 (50.7, 51.5) 48.9 (48.5, 49.3)	38.4 (32.5, 44.8) 61.6 (55.2, 67.5)	<0.001
Equivilised household income, % #	Below the poverty line Above the poverty line	18.1 (17.0, 19.2) 81.9 (80.8, 83.0)	44.7 (37.7, 51.7) 55.3 (48.3, 62.3)	<0.001
Country of birth, % *	Australia English speaking Non-English speaking	68.6 (66.9, 70.3) 11.7 (10.8, 12.6) 19.7 (18.1, 21.4)	74.4 (67.7, 80.2) 10.0 (5.7, 17.0) 15.6 (10.5, 22.5)	0.33
Location, % ±	Major city Inner regional Outer regional/remote	71.7 (70.5, 73.0) 19.0 (17.3, 20.8) 9.3 (7.8, 11.0)	66.0 (59.6, 71.7) 24.0 (18.6, 30.3) 10.1 (6.8, 14.6)	0.11
Labour status, %	Employed Unemployed Not in labour force	67.7 (66.3, 69.1) 2.3 (1.9, 2.9) 30.0 (28.8, 31.2)	49.7 (42.7, 56.7) 9.4 (5.5, 15.6) 40.9 (34.4, 47.8)	<0.001
Marital status, %	Married Not married	60.1 (58.9, 61.3) 39.9 (38.7, 41.1)	33.8 (27.0) 66.2 (58.7, 73.0)	<0.001
Educational obtainment, % ^^	Low Medium High	25.4 (24.2, 26.5) 49.2 (47.6, 50.8) 25.5 (24.1, 26.9)	37.9 (31.0, 45.4) 47.9 (40.3, 55.6) 14.2 (9.5, 20.7)	<0.001
Body Mass Index, % ##	Underweight Normal weight Overweight Obese	2.2 (1.7, 2.7) 34.0 (32.6, 35.4) 36.6 (35.1, 38.1) 27.2 (25.9, 28.6)	2.9 (0.4, 5.4) 35.6 (28.8, 42.4) 27.5 (21.0, 33.9) 33.9 (27.5, 40.4)	0.06
Smoking status, % **	Current smoker Ex-smoker Never smoked	16.8 (15.7, 17.9) 31.4 (30.1, 32.8) 51.8 (50.3, 53.2)	50.3 (42.6, 58.0) 23.8 (18.2, 30.5) 25.9 (20.8, 31.8)	<0.001

Values represent mean and in brackets, 95% confidence intervals after applying survey weighting, rounded to 1 decimal place. ^ Unadjusted survey-weighted regression models were used to compare characteristics between food-secure and food-insecure populations. # Below poverty line—income decile 1 and 2 ≤ AUD 398/weekly; above poverty line—income decile 3–10. * English Speaking—Canada, Repub. of Ireland, New Zealand, South Africa, UK and USA. We considered anglophone countries to have some similarities in terms of food cultures and diets, and hence they were grouped together. ± Outer regional/remote—outer regional Australia, remote Australia and very remote Australia, ^^ Low—incomplete high school or less; medium—completed high school or incomplete high school and/or certificate/diploma; high—tertiary qualification. ## Body mass Index (BMI). Underweight is BMI less than 18.5 kg/m^2^, normal BMI is 18.5 to < 25 kg/m^2^, overweight BMI is 25.0 to <30 kg/m^2^, Obese BMI is 30.0 kg/m^2^ or higher ** Current—daily, at least once a week and less than weekly.

**Table 2:** DGI total and component scores by food security status.

DGI Component Mean Scores *	Food-Secure	Food-Insecure	Model 1—Group Differences (Minimally Adjusted) ^a^	
	Mean (95% CI)	Mean (95% CI)	B (95% CI)	*p* Value
1. Food variety	2.3 (2.3, 2.3)	1.6 (1.5, 1.8)	−0.54 (−0.72, −0.36)	<0.005
2. Fruit	5.0 (4.9, 5.1)	3.8 (3.1, 4.5)	−1.06 (−1.69, −0.43)	0.001
3. Vegetables	4.4 (4.3, 4.5)	3.7 (3.2, 4.2)	−0.62 (−1.09, −0.15)	0.010
4. Cereal (total) serves per day	2.8 (2.8, 2.9)	2.4 (2.2, 2.7)	−0.26 (−0.50, −0.03)	0.026
mostly wholegrain	1.5 (1.4, 1.5)	1.0 (0.8, 1.2)	−0.31 (−0.57, −0.05)	0.021
5. Meat and alternatives (total) serves per day	3.0 (2.9, 3.0)	2.6 (2.3, 2.9)	−0.28 (−0.55, −0.01)	0.045
mostly lean	4.5 (4.4, 4.5)	4.5 (4.3, 4.6)	0.01 (−0.18, 0.19)	0.95
6. Dairy and alternatives (total)	4.7 (4.6, 4.8)	4.6 (4.1, 5.1)	−0.32 (−0.80, 0.16)	0.19
7. Fluid intake (total) serves per day	3.8 (3.7, 3.8)	3.9 (3.6, 4.1)	−0.02 (−0.21, 0.18)	0.87
mostly water	4.3 (4.3, 4.4)	4.4 (4.2, 4.6)	−0.01 (−0.20, 0.17)	0.90
8. Limit discretionary foods	3.3 (3.1, 3.4)	3.5 (2.8, 4.2)	0.21 (−0.42, 0.84)	0.51
9. Limit saturated fat mostly trimmed meat	4.4 (4.4, 4.5)	4.2 (3.9, 4.5)	−0.22 (−0.47, 0.03)	0.08
mostly low-fat milk	3.8 (3.7, 3.9)	3.7 (3.4, 4.0)	−0.02 (−0.33, 0.29)	0.91
10. Moderate unsaturated fat	8.2 (8.0, 8.3)	8.7 (8.1, 9.3)	0.60 (0.10, 1.09)	0.018
11. Limit added salt during cooking	2.6 (2.5, 2.6)	2.5 (2.1, 2.8)	−0.02 (−0.34, 0.29)	0.88
at the table	3.3 (3.2, 3.4)	2.8 (2.5, 3.2)	−0.58 (−0.89, −0.27)	<0.001
12. Limit extra sugars	6.6 (6.4, 6.7)	6.5 (5.9, 7.0)	0.13 (−0.49, 0.76)	0.68
13. Limit alcohol	8.5 (8.4, 8.6)	8.5 (8.1, 9.0)	−0.20 (−0.71, 0.31)	0.45
TOTAL DGI SCORE	76.8 (76.3, 77.3)	72.8 (70.5, 75.2)	−3.52 (−5.57, −1.46)	0.001

Values represent unadjusted means and in brackets, 95% confidence intervals after applying survey weighting * See Supplement Table S1 for how scores are calculated. ^a^ Mean difference between food-secure and food-insecure groups, adjusted for age and sex, estimated using survey-weighted multiple imputation linear regression models.

**Table 3:** Total energy and nutrient intakes of adults by food security status.

Energy/Nutrient Mean	Food-Secure	Food-Insecure	Model 1—Group Differences (Minimally Adjusted) ^a^	
	Mean (95% CI)	Mean (95% CI)	B (95% CI)	*p* Value
Energy intake (kJ/day)	8694 (8589, 8800)	8147 (7622, 8672)	−474.2 (−943.3, −5.1)	0.048
Protein intake (%E/day)	18.5 (18.3, 18.6)	17.2 (16.3, 18.1)	−1.24 (−2.09, −0.39)	0.004
Total fat (%E/day)	30.9 (30.6, 31.1)	30.0 (28.5, 31.4)	−1.13 (−2.42, 0.17)	0.09
Saturated fat intake (%E/day)	12.1 (11.9, 12.2)	12.1 (11.4, 12.8)	−0.01 (−0.74, 0.72)	0.97
Trans fat intake (%E/day)	0.5 (0.6, 0.6)	0.6 (0.5, 0.6)	0.01 (−0.05, 0.07)	0.81
Mono-unsaturated fat intake (%E/day)	11.8 (11.7, 12.0)	11.2 (10.5, 11.9)	−0.73 (−1.37, −0.09)	0.026
Poly-unsaturated fat intake (%E/day)	4.8 (4.7, 4.9)	4.6 (4.2, 5.0)	−0.29 (−0.66, 0.08)	0.13
Carbohydrate intake (%E/day)	43.3 (43.0, 43.6)	45.6 (44.0, 47.2)	2.03 (0.65, 3.41)	0.004
Total sugars intake (%E/day)	19.0 (18.8, 19.3)	21.8 (19.8, 23.8)	2.55 (0.90, 4.21)	0.003
Fibre intake (g/MJ)	23.0 (22.6, 23.4)	20.1 (18.3, 21.8)	−2.29 (−3.81, −0.77)	0.003
Sodium intake (mg/MJ)	2443 (2407, 2479)	2321 (2152, 2490)	−136.7 (−321.6, 48.2)	0.15

Values represent unadjusted means and in brackets, 95% confidence intervals after applying survey weighting. ^a^ Mean difference between food-secure and food-insecure groups, adjusted for age and sex, estimated using survey weighted multiple imputation linear regression models.

## Data Availability

Data are available from the Australian Bureau of Statistics.

## Supplementary Materials

The following are available online at https://www.mdpi.com/article/10.3390/nu14194133/s1, Table S1. Components and scoring methods of the Dietary Guideline Index (DGI-2013). Table S2. DGI score by food security status, Table S3. Total energy and nutrient intakes of adults by food security status.
