# Supplementary material for: The Diet Quality of Food-Insecure Australian Adults—A Nationally Representative Cross-Sectional Analysis
Source: Nutrients. 2022 Oct 5;14(19):4133. doi: 10.3390/nu14194133 (PMC9572719; doi:10.3390/nu14194133)
Supplement: Supplementary file 1 [file nutrients-14-04133-s001.zip › Revised Supplement 1 .pdf]

Table S1. Components and scoring methods of the Dietary Guideline Index (DGI-2013)

Dietary Guideline	Indicator and description	Criteria for maximum score[1]	Criteria for minimum score (0)	Maximum Score
Guidelines for adequate intake				
1. Enjoy a wide variety of nutritious foods	Food variety[2]: proportion of food from each of the 5 core food groups eaten at least one serve per week	100%	0%	10
2. Plenty of vegetables	Total vegetable intake: servings of vegetables per day	19-50 y: M ≥ 6, F ≥ 5<br>51-70 y: M ≥ 5.5, F ≥ 5<br>>70 y: M ≥ 5, F ≥ 5	0	10
3. Fruit	Total fruit intake: servings of fruit per day	≥ 2	0	10
4. Grain (cereal) foods	Total cereal intake: servings of grains per day	19-50 y: M ≥ 6, F ≥ 6<br>51-70 y: M ≥ 6, F ≥ 4<br>>70 y: M ≥ 4.5, F ≥ 3	0	5
	Wholegrain or high fiber cereals: proportion of wholegrain bread to white bread intake per day	≥ 50% wholemeal bread	0%	5
5. Lean meat and poultry, fish, eggs, nuts and seeds, and legumes/beans	Total lean meat and alternative: servings per day	19-50 y: M ≥ 3, F ≥ 2.5<br>51-70 y: M ≥ 2.5, F ≥ 2<br>>70 y: M ≥ 2.5, F ≥ 2	0	5
	Lean meat: proportion of lean meats and alternatives to total meat and alternatives per day	100%	0%	5
6. Milk, yoghurt, cheese and/or their alternatives[3]	Total dairy and alternative: servings per day	19-50 y: M ≥ 2.5, F ≥ 2.5<br>51-70 y: M ≥ 2.5, F ≥ 4<br>>70 y: M ≥ 3.5, F ≥ 4	0	10
7. Drink plenty of water	Total beverage intake[4]: servings per day	M ≥ 10; F ≥ 8	0	5
	Water[5]: proportion of water to total beverage intake per day	≥ 50%	0%	5
Guidelines to limit or moderate intake				
8. Limit intake of foods containing saturated fat, added salt, added sugars and alcohol	Limit discretionary foods	M ≤ 3; F ≤ 2.5	M > 3; F > 2.5	10
9. Limit intake of foods high in saturated fat	Trim meat: proportion of trimmed meat to total meat	≥ 50%	0%	5
	Choose reduced-fat milk: proportion of reduced-fat milk to total milk intake per day	≥ 50% reduced-fat milk	0%	5
10. Small allowance of unsaturated oils, fats or spreads	Unsaturated spreads and oils: servings per day	19-50 y: M ≤ 4, F ≤ 2<br>51-70 y: M ≤ 4, F ≤ 2<br>>70 y: M ≤ 2, F ≤ 2	M > 4; F > 2	10
11. Limit intake of foods and drinks containing added salt	Salt use: salt added during cooking	Not used or rarely	Very often	5
	Salt use: salt added during the meal	Not used or rarely	Very often	5
12. Limit intake of foods and drinks containing added sugars	Limit extra sugar[6]: servings per day	M ≤ 1.5; F ≤ 1.25	M > 1.5; F > 1.25	10
13. If you choose to drink alcohol, limit intake	Limit alcohol: servings per day	≤ 2	> 2	10

1. Criteria for maximum scores were derived from the Australian Dietary Guidelines 2013 unless otherwise noted.
2. Food variety was measured and scored using a similar method to the Recommended Food Score.
3. Choosing reduced fat dairy is captured in the "Limit intake of foods high in saturated fat" component.
4. The Eat for Health Australian Dietary Guidelines do not have specific recommendations for beverage consumption and recommended the guidelines found in the Nutrient Reference Values for Australia and New Zealand.
5. The proportion of water to total beverage intake was derived from US beverage guidelines.
6. Since added sugar intake is not recommended there are no cut-off values for number or recommended serves, instead half of the maximum discretionary food cut-off were used consistent with the original DGI.

Reproduced with permission from M Thorpe – reference Thorpe MG, Milte CM, Crawford D, McNaughton SA. A Revised Australian Dietary Guideline Index and Its Association with Key Sociodemographic Factors, Health Behaviors and Body Mass Index in Peri-Retirement Aged Adults. Nutrients. 2016;8(3):160.
