# Supplementary material for: The Diet Quality of Food-Insecure Australian Adults—A Nationally Representative Cross-Sectional Analysis
Source: Nutrients. 2022 Oct 5;14(19):4133. doi: 10.3390/nu14194133 (PMC9572719; doi:10.3390/nu14194133)
Supplement: Supplementary file 1 [file nutrients-14-04133-s001.zip › Supplement 2.pdf]

Table S2. DGI score by food security status

DGI Component mean scores*	Food secure	Food insecure	Model 2[B]	
	**Mean (95% CI)**	**Mean (95% CI)**	**B (95% CI)**	**p value**
**1.** Food variety	2.3 (2.3, 2.3)	1.6 (1.5, 1.8)	-0.24 (-0.41, -0.06)	0.009
2. Fruit	5.0 (4.9, 5.1)	3.8 (3.1, 4.5)	-0.26 (-0.88, 0.36)	0.42
3. Vegetables	4.4 (4.3, 4.5)	3.7 (3.2, 4.2)	-0.37 (-0.84, 0.10)	0.12
4. Cereal (total)	2.8 (2.8, 2.9)	2.4 (2.2, 2.7)	-0.18 (-0.42, 0.05)	0.13
serves per day				
mostly wholegrain	1.5 (1.4, 1.5)	1.0 (0.8, 1.2)	-0.17 (-0.44, 0.10)	0.21
5. Meat & alternatives (total)	3.0 (2.9, 3.0)	2.6 (2.3, 2.9)	-0.12 (-0.39, 0.16)	0.40
serves per day				
mostly lean	4.5 (4.4, 4.5)	4.5 (4.3, 4.6)	0.09 (-0.10, 0.28)	0.34
6. Dairy & alternatives (total)	4.7 (4.6, 4.8)	4.6 (4.1, 5.1)	0.00 (-0.49, 0.48)	0.99
7. Fluid intake	3.8 (3.7, 3.8)	3.9 (3.6, 4.1)	0.09 (-0.11, 0.29)	0.39
serves per day				
mostly water	4.3 (4.3, 4.4)	4.4 (4.2, 4.6)	0.00 (-0.19, 0.19)	0.98
8. Limit discretionary foods	3.3 (3.1, 3.4)	3.5 (2.8, 4.2)	0.49 (-0.16, 1.14)	0.14
9. Limit saturated fat	4.4 (4.4, 4.5)	4.2 (3.9, 4.5)	-0.12 (-0.37, 0.12)	0.32
mostly trimmed meat				
mostly low fat milk	3.8 (3.7, 3.9)	3.7 (3.4, 4.0)	0.04 (-0.27, 0.36)	0.79
10. Moderate unsaturated fat	8.2 (8.0, 8.3)	8.7 (8.1, 9.3)	0.36 (-0.15, 0.88)	0.17
11. Limit added salt	2.6 (2.5, 2.6)	2.5 (2.1, 2.8)	0.03 (-0.29, 0.34)	0.86
during cooking				
at the table	3.3 (3.2, 3.4)	2.8 (2.5, 3.2)	-0.33 (-0.63, -0.03)	0.030
12. Limit extra sugars	6.6 (6.4, 6.7)	6.5 (5.9, 7.0)	0.19 (-0.45, 0.83)	0.56
13. Limit alcohol	8.5 (8.4, 8.6)	8.5 (8.1, 9.0)	0.05 (-0.46, 0.57)	0.84
TOTAL DGI SCORE	76.8 (76.3, 77.3)	72.8 (70.5, 75.2)	-0.45 (-2.48, 1.58)	0.66

Values represent mean and in brackets, 95% confidence intervals after applying survey weighting, rounded to 1 decimal place *See supplement Table 1 for how scores are calculated

[b] Mean difference between food secure and food insecure groups, adjusted for age, sex, equivalized household income, educational obtainment, country of birth, marital and smoking status, estimated using survey weighted multiple imputation linear regression models.

Table S3. Total energy and nutrient intakes of adults by food security status

Energy/nutrient mean	Food secure (n=8716)	Food insecure (n=399)	Model 2[B]	
	**Mean (95% CI)**	**Mean (95% CI)**	**B (95% CI)**	**P value**
Energy intake (kJ/day)	8694 (8589, 8800)	8147 (7622, 8672)	-320.3 (-797.3, 156.6)	0.19
Protein intake (%E/day)	18.5 (18.3, 18.6)	17.2 (16.3, 18.1)	-0.98 (-1.86, -0.10)	0.030
Total fat (%E/day)	30.9 (30.6, 31.1)	30.0 (28.5, 31.4)	-1.26 (-2.61, 0.10)	0.069
Saturated fat intake (%E/day)	12.1 (11.9, 12.2)	12.1 (11.4, 12.8)	-0.42 (-1.16, 0.33)	0.27
Trans fat intake (%E/day)	0.5 (0.6, 0.6)	0.6 (0.5, 0.6)	-0.03 (-0.09, 0.03)	0.40
Mono-unsaturated fat intake (%E/day)	11.8 (11.7, 12.0)	11.2 (10.5, 11.9)	-0.73 (-1.39, -0.07)	0.031
Poly-unsaturated fat intake (%E/day)	4.8 (4.7, 4.9)	4.6 (4.2, 5.0)	-0.07 (-0.44, 0.31)	0.73
Carbohydrate intake (%E/day)	43.3 (43.0, 43.6)	45.6 (44.0, 47.2)	2.28 (0.89, 3.66)	0.001
Total sugars intake (%E/day)	19.0 (18.8, 19.3)	21.8 (19.8, 23.8)	2.28 (0.59, 3.97)	0.008
Fibre intake (g/MJ)	23.0 (22.6, 23.4)	20.1 (18.3, 21.8)	-0.34 (-1.86, 1.17)	0.66
Sodium intake (mg/MJ)	2442.8 (2406.6, 2478.9)	2320.5 (2151.5, 2489.5)	-132.2 (-321.8, 57.4)	0.17

Values represent mean and in brackets, 95% confidence intervals after applying survey weighting, rounded to 1 decimal place

[b] Mean difference between food secure and food insecure groups, adjusted for age, sex, equivalized household income, educational obtainment, country of birth, marital and smoking status (categorical), estimated using survey weighted multiple imputation linear regression models.
